# Monitoring of cyanobacterial breakthrough and accumulation by in situ phycocyanin probe system within full-scale treatment plants

**DOI:** 10.1007/s10661-023-11657-0

**Published:** 2023-08-17

**Authors:** Liya Ma, Juan Francisco Guerra Maldonado, Arash Zamyadi, Sarah Dorner, Michèle Prévost

**Affiliations:** 1 Department of Civil, Geological, and Mining Engineering, Polytechnique Montreal, Montreal, QC H3C 3A7 Canada; 2 Department of Civil Engineering, Monash University, Clayton Campus, Melbourne, Australia

**Keywords:** Cyanobacteria, Phycocyanin, Fluorescence probe, Water treatment plant

## Abstract

Worldwide, there has been an increase in the presence of potentially toxic cyanobacterial blooms in drinking water sources and within drinking water treatment plants (DWTPs). The objective of this study is to validate the use of in situ probes for the detection and management of cyanobacterial breakthrough in high and low-risk DWTPs. In situ phycocyanin YSI EXO2 probes were devised for remote control and data logging to monitor the cyanobacteria in raw, clarified, filtered, and treated water in three full-scale DWTPs. An additional probe was installed inside the sludge holding tank to measure the water quality of the surface of the sludge storage tank in a high-risk DWTP. Simultaneous grab samplings were carried out for taxonomic cell counts and toxin analysis. A total of 23, 9, and 4 field visits were conducted at the three DWTPs. Phycocyanin readings showed a 93-fold fluctuation within 24 h in the raw water of the high cyanobacterial risk plant, with higher phycocyanin levels during the afternoon period. These data provide new information on the limitations of weekly or daily grab sampling. Also, different moving averages for the phycocyanin probe readings can be used to improve the interpretation of phycocyanin signal trends. The in situ probe successfully detected high cyanobacterial biovolumes entering the clarification process in the high-risk plant. Grab sampling results revealed high cyanobacterial biovolumes in the sludge for both high and low-risk plants.

**Supplementary Information:**

The online version contains supplementary material available at 10.1007/s10661-023-11657-0.

## Introduction

In recent years, an increase in the occurrence of potentially toxic cyanobacterial blooms has been reported in water supply systems worldwide (Akyol et al., 2021; Kim & Park, 2021; Zamyadi et al., 2019). Climate change and human activities may intensify the frequency and duration of harmful cyanobacterial blooms (Griffith & Gobler, 2020; Nwankwegu et al., 2019). Concerns related to the impacts of cyanobacteria in water supply systems are related to human health risks (e.g., skin diseases, gastroenteritis, Alzheimer’s disease, and liver damage) and esthetic water quality (e.g., taste and odor) (Chernoff et al., 2017; Chorus & Welker, 2021; Kim & Park, 2021). Due to such effects of toxic cyanobacterial blooms, several monitoring frameworks were developed over the last two decades to aid the management of cyanobacterial blooms in drinking water supplies (Chorus & Welker, 2021; Ellis, 2009; Ministry of Health, 2020; NHMRC, 2022).

The action plans based on the different alert levels consist of weekly grab sampling once the waterbody or raw water has been tested for positive toxins or potentially toxigenic cyanobacteria (Chorus & Welker, 2021; Ministry of Health, 2020). The grab sampling frequency is not standardized. To the authors’ knowledge, the highest sampling frequency suggested by management strategies is twice per week (Newcombe et al., 2010). Such recommended monitoring frequencies may not be appropriate to estimate the cyanobacterial concentration in the source water and inside the drinking water treatment plants (DWTPs), since the bloom conditions may change rapidly, and the sampling schedules did not always correspond to the peak bloom period (Genzoli & Kann, 2016; Zamyadi et al., 2014). Furthermore, the processing time needed by laboratory taxonomic cell counts and toxin analyses could contribute to a longer response time. These limitations are critical for the cyanobacteria management for water utilities, given the need for prompt intervention and management to prevent the presence and accumulation of cyanobacteria inside the DWTPs. Thus, intensive in situ fluorescence measurement tools for the rapid detection of cyanobacteria-specific pigment, i.e., phycocyanin, have been increasingly used for cyanobacterial quantification. Besides the high detention frequency, the advantages of the in situ phycocyanin probes are inexpensive and operational simplicity (Bertone et al., 2018; Zamyadi et al., 2016).

Extensive studies have demonstrated the application of the in situ phycocyanin fluorescence probe to prevent the entry of cyanobacterial cells within the DWTPs by source water or raw water monitoring (Bowling et al., 2016; Cotterill et al., 2019; Gregor et al., 2007; Izydorczyk et al., 2009; McQuaid et al., 2011; Thomson-Laing et al., 2020). However, there are limited applications of using the probe within the full-scale DWTPs. The occurrence of blooms may affect the cyanobacterial removal efficiency, leading to cyanobacteria and cyanotoxins breakthrough into various processes of DWTPs. Reported instances of cyanobacteria accumulation were observed in filter media, surface of clarifier, surface of filter, sludge thickener, and supernatant of sludge thickener (Jalili et al., 2021; Pestana et al., 2016; Zamyadi et al., 2013a, 2019). Almuhtaram et al. (2018) measured the phycocyanin fluorescence of the raw water, surface of clarifier and filter, and treated water at four Great Lakes region DWTPs. But the measurements were only recorded at each sampling visit. Such discrete probe monitoring frequencies compromise the online assessment and were insufficient to identify the intermittent cyanobacterial breakthrough within the DWTPs. Zamyadi et al. (2013b) and (2014) used online probe data throughout the treatment process, but only 1 data were recorded every 4 h. Such frequency may not capture the actual cyanobacterial dynamic, due to the hypothesized highly temporal fluctuation of the cyanobacteria biomass in the water source. In addition, the phycocyanin level for the surface of the sludge holding tank was not measured by the in situ probe from these studies (Almuhtaram et al., 2018; Zamyadi et al., 2013b, 2014). This study further explored the applicability of phycocyanin probes when applied across the treatment train in both high and low cyanobacterial risk DWTPs and provides a quantitative evaluation of cyanobacterial cell accumulation and breakthrough, allowing the operators to use the probe to evaluate the need for treatment adjustments.

The main objective of this work is to apply time series analysis on continuous phycocyanin probe data to investigate cyanobacteria dynamics inside the high- and low-risk full-scale DWTPs, including bloom timing and magnitude at various locations within the DWTPs. The specific objectives are to (1) investigate the use of in situ probe readings to track cyanobacterial fluctuations in DWTPs on daily and hourly basis and (2) evaluate in situ probes for mapping and identifying the critical points for breakthrough and accumulation of cells and their associated toxins using a combination of probe readings and taxonomic cell counts.

## Materials and methods

### Water source and site description

Sampling campaigns were undertaken at three full-scale DWTPs in Canada (Fig. S1) during the summer and fall of 2018. The three DWTPs can provide the opportunity to study both low and high-risk incoming cyanobacterial cells into their intake water. The treatment processes in the three plants are listed in Table S1.

**Fig. 1:**
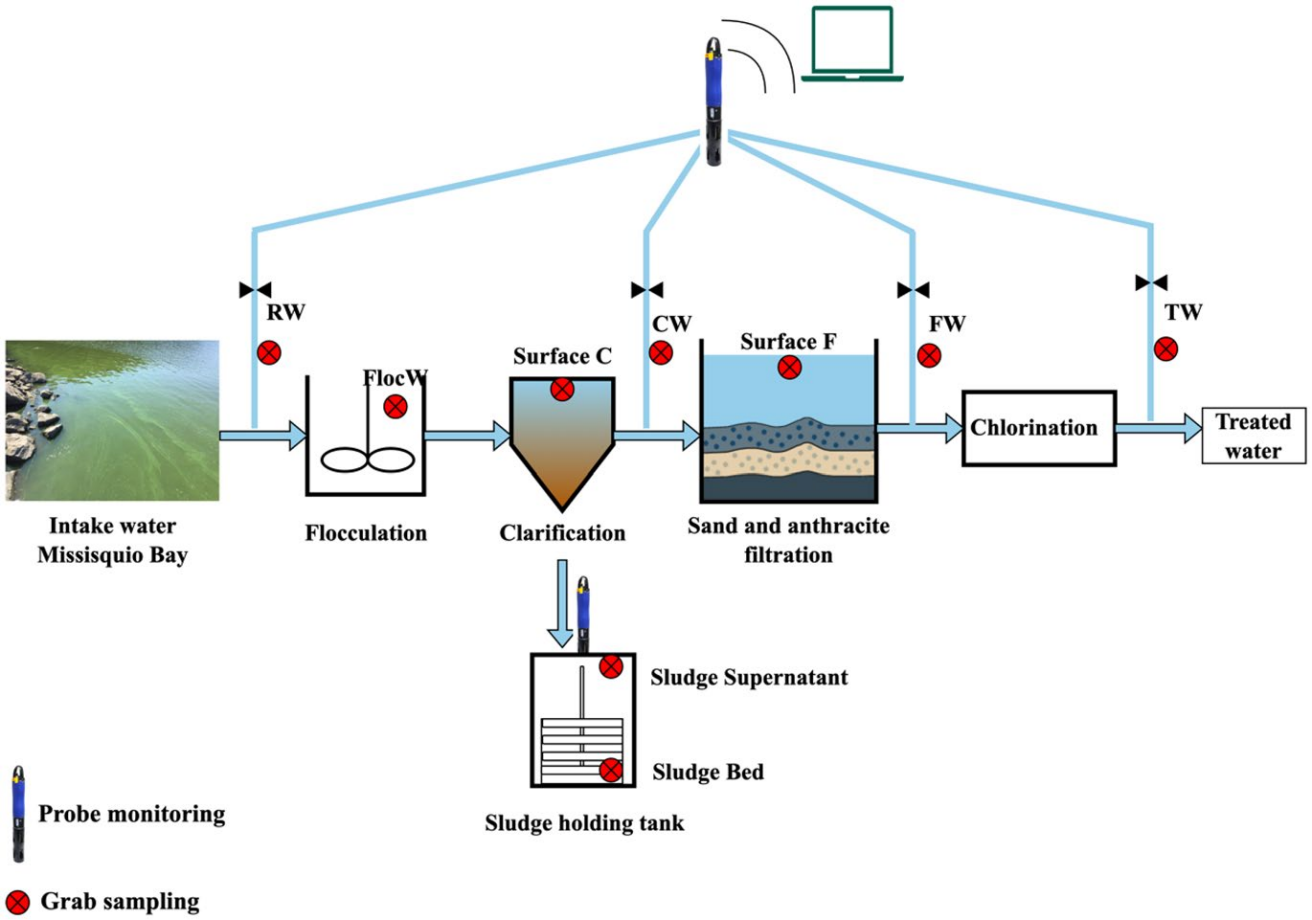
Schematic of the treatment processes of DWTP A, grab sampling, and probe monitoring locations. The water intake is from Missisquoi Bay. Sampling points are indicated by raw water (RW), flocculated water (FlocW), clarified water (CW), filtered water (FW), treated water (TW), surface of clarifier (Surface C), surface of filter (Surface F), supernatant of sludge storage tank (Sludge Supernatant), and sludge bed of sludge holding tank (Sludge Bed)

DWTP A is located on Missisquoi Bay of Lake Champlain. Due to the high concentration of phosphorus and nitrogen in the bay, *Microcystis* cyanobacterial blooms occur during the summer and fall in recent decades (Jalili et al., 2021; McQuaid et al., 2011). The plant employs conventional treatment trains as presented in Fig. 1. The processes are powdered activated carbon (PAC) injection followed by coagulation, flocculation, and sedimentation, then chlorination. The sludge supernatant is discharged into the lake, while the sludge is transferred to the wastewater treatment plant. The source water is directed into the raw water tank prior to treatment.

DWTP B draws water from Lemieux reservoir which is fed by the North Yamaska River. The Yamaska River is classified as eutrophic (Remmal et al., 2017). Lime is added to the reservoir prior to treatment to control the pH. Solar mixers are installed in the reservoir to prevent the creation and proliferation of cyanobacteria. An overview of the treatment processes of DWTP B and the location of sampling points is illustrated in Fig. S2.

DWTP C is in Southwest Ontario. It supplies potable water for Leamington, Lakeshore, Kingsville, and Essex. The plant draws water from Lake Erie, and the water is pre-chlorinated before entering the plant. During the summer bloom season, the cyanobacteria start forming in the shallow warm waters of Lake Erie close to Ohio and then are transported throughout Western Lake Erie. Cyanobacterial blooms have been observed annually in recent years in Lake Erie (Jankowiak et al., 2019). An overview of the treatment processes of DWTP C and the location of sampling points is illustrated in Fig. S3.

### In situ phycocyanin monitoring

YSI EXO2 water quality multi-probe (YSI, Yellow Springs, OH, USA) fitted with in situ phycocyanin fluorescence sensor was used in this study. Phycocyanin optics has the excitation and emission wavelength of 590 ± 15 nm and 685 ± 20 nm, respectively (YSI Incorporated, 2019). In each of the three studied DWTPs, a YSI EXO2 probe with its remote control and data logging was installed to measure the phycocyanin RFU of raw water (RW), clarified water (CW), filtered water (FW), and treated water (TW). Each probe was equipped with a self-cleaning wiper. In-plant samples were almost not exposed to light, and the flow chamber of the probe has a black bottom to prevent light influence. Figure S4 demonstrates the probe monitoring system. The switches are used for controlling the electronic solenoids, so that RW, CW, FW, and TW can continuously go through the probe flow cell, separately. Four types of water went through the probe flow cell sequentially (30 min cycle), and probe readings were recorded at 5-min intervals. Therefore, for each water type, six measurements were recorded every 2 h. The flow rates through the probe flow cell were adjusted to ensure the absence of air bubbles in the probe cell and to fully flush the probe cell when switching between water types. Probe data were continuously recorded, transmitted via a modem, and downloaded remotely. Negative phycocyanin readings which represent readings out of the probe’s detection limit were replaced with zero.

For DWTP A, an additional YSI EXO2 phycocyanin probe was installed inside the sludge holding tank to measure the water quality of the supernatant of sludge storage tank (Sludge Supernatant), as shown in Fig. 1.

### Sampling procedure and analysis of water quality parameters

From July to October of 2018, a total of 23, 9, and 4 field visits were conducted at A, B, and C DWTPs, respectively. These visits were conducted to calibrate the probe and collect grab samples. Grab samples were taken in parallel with probe measurements from the RW, CW, FW, and TW from all three DWTPs. They were collected from sampling taps available inside the DWTPs and were fully flushed before collection.

In addition, targeted grab samples were also collected at several sites throughout the DWTPs to evaluate the distribution of cyanobacteria levels and the treatment performance. The additional grab sample sites were different within each DWTP and are listed as follows.

For DWTP A: flocculated water (FlocW), apparent accumulation at the surface of clarified water (Surface C), apparent accumulation at the surface of filtered water (Surface F), supernatant of sludge storage tank (Sludge Supernatant), and the underlying sludge bed of sludge holding tank (Sludge Bed). For DWTP B: underlying sludge bed of sludge holding tank (Sludge Bed). For DWTP C: apparent accumulation at the surface of clarified water (Surface C), apparent accumulation at the surface of filtered water (Surface F), and underlying sludge bed of sludge holding tank (Sludge Bed).

Taxonomic cell counts (in singlicate) and toxin analysis (in duplicate) were undertaken on all samples. The taxonomic cell count samples were preserved with Lugol’s iodine in the dark at room temperature (21 °C). The analyses of taxonomic counts, species identification, and biovolumes calculation were achieved at Université du Québec à Montréal’s (UQAM) Biological Sciences Department. The method is according to Lund (1959) and Planas et al. (2000). The reproducibility of taxonomic cell counts has been discussed by Zamyadi et al. (2012) and was conducted with the same protocol and technician as in our study.

With regards to the microcystin (MC) analyses, an on-line solid-phase extraction ultra-high-performance liquid chromatography coupled to tandem mass spectrometry (On-line SPE-UHPLC-MS/MS, Thermo TSQ Quantiva) was used to determine the total MC concentrations. More details on the cyanotoxin analysis methods are explained in Munoz et al. (2017) and Roy-Lachapelle et al. (2019).

### Data analysis

Statistical analysis was done using R (version 4.2.1). Moving average statistics were calculated using the rollmean function in the zoo package (version 1.8.10). ANOVA was performed with the factor of the hour of the day, and Tukey’s HSD test was used as the post hoc multiple comparison, with the significance level *α* = 0.1.

## Results

### Time series analysis of the intake water of a high-risk DWTP

**Fig. 2:**
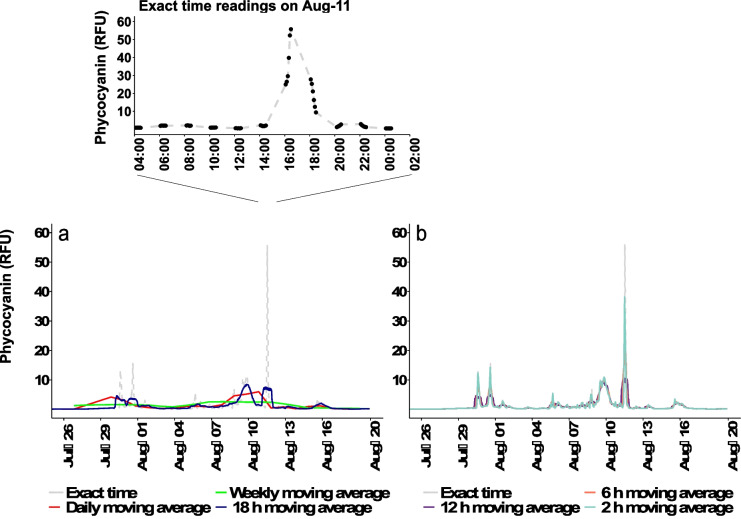
Temporal monitoring of different time-series analysis of in situ YSI EXO2 phycocyanin probe readings (RFU) at the raw water intake of the DWTP A during July and August of 2018. **a** Exact time, daily, weekly, and 18-h moving averages; **b** exact time, 2, 6, and 12-h moving averages

Individual phycocyanin fluorescence readings varied greatly within a 24-h period during the 32 days of monitoring in July and August 2018 (Fig. 2). Quick and substantial fluctuations can occur as shown on August 11, when phycocyanin readings increased 93-fold, from 0.6 RFU at 12:30 to 55.8 RFU at 16:30 based on raw probe measurements (exact time), exceeding the mean RFU reading of 1.1 RFU for the whole period. Specifically, phycocyanin values over 1.8 RFU equivalent (calculated from the site-specific equivalency derived from McQuaid et al. (2011) to the Alert Level 1 of Chorus and Welker (2021)) were measured in 13.5% of the readings on 11/32 days of monitoring. Readings exceeding 5.0 RFU (calculated from the site-specific equivalency derived from McQuaid et al. (2011) to the Alert Level 2 of Chorus and Welker (2021)) were less common and of shorter duration. Values exceeding 5.0 RFU were measured in 5.0% of the readings on 7/32 days of monitoring. Notably, the longer-lasting high values all occurred during the afternoon period.

As probes produce high frequency readings, the interpretation of measurements could be facilitated by using daily moving average, weekly moving average, 2, 6, 12, and 18-h moving averages as shown in Fig. 2. During the monitoring period, the phycocyanin readings varied within the range of 0–55.8 RFU using raw measurements. The highest readings decreased by using moving averages. For instance, the highest phycocyanin reading was 38.1 RFU using 2-h moving average, and the highest phycocyanin reading was 5.6 RFU using daily moving average.

**Fig. 3:**
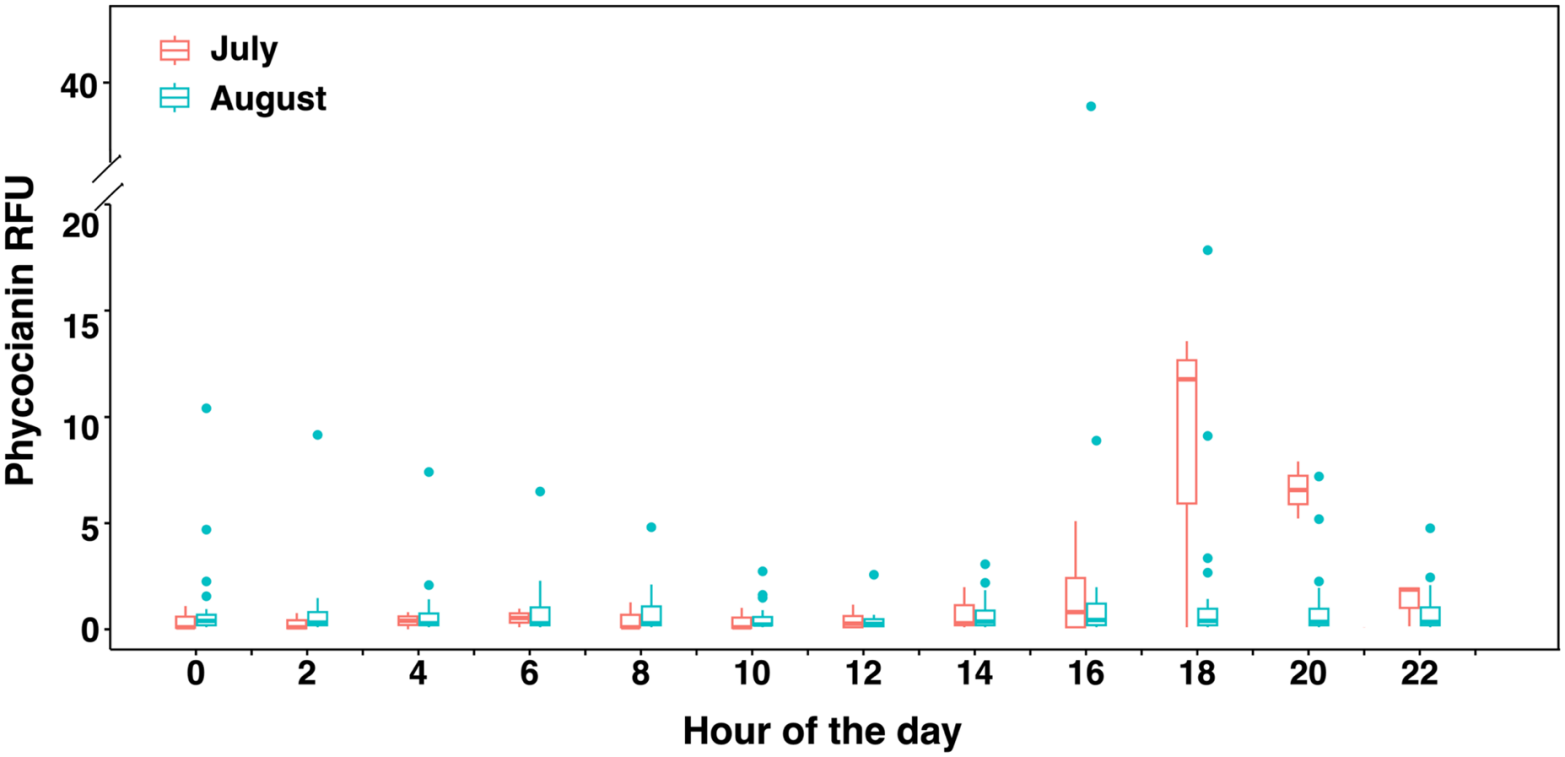
Phycocyanin (RFU) by hour of the day at DWTP A raw water intake during July (*n* = 7 per hour) and August (*n* = 25 per hour) of 2018. The bottom and top of each box represent the 25–75th percentiles, respectively. The whiskers represent the minimum and maximum values. The line within each box corresponds to the median value

To investigate whether high phycocyanin readings are observed during some periods of the day, phycocyanin readings were sorted per hour over a 7-day period in July and a 25-day period in August (Fig. 3). Higher mean phycocyanin values were observed during the afternoon period (after 16 h), for both July and August. ANOVA tests show statistically significant differences in phycocyanin readings during the different hours of the day during July, and Tukey’s HSD post hoc tests reveal significant differences in the comparisons of 18 h and the other measured time (Fig. S5).

Additionally, the correlations between total cyanobacterial biovolumes and the probe readings were investigated (Fig. S6). As multiple flows were measured by the probe, the phycocyanin readings for the raw water were recorded 30 min every 2 h with 5 min intervals, so different time-series statistics of the phycocyanin readings were considered. The readings most closely associated with the 2-h moving average (*R* = 0.73, *p* = 0.098) to account for the differences in the timing of grab sampling and probe readings.

### Applying the probe readings to estimate the risk of cyanobacteria within a high-risk DWTP

#### Cyanobacterial cells and cyanotoxins in the RW, CW, FW, and TW in a high-risk DWTP

**Fig. 4:**
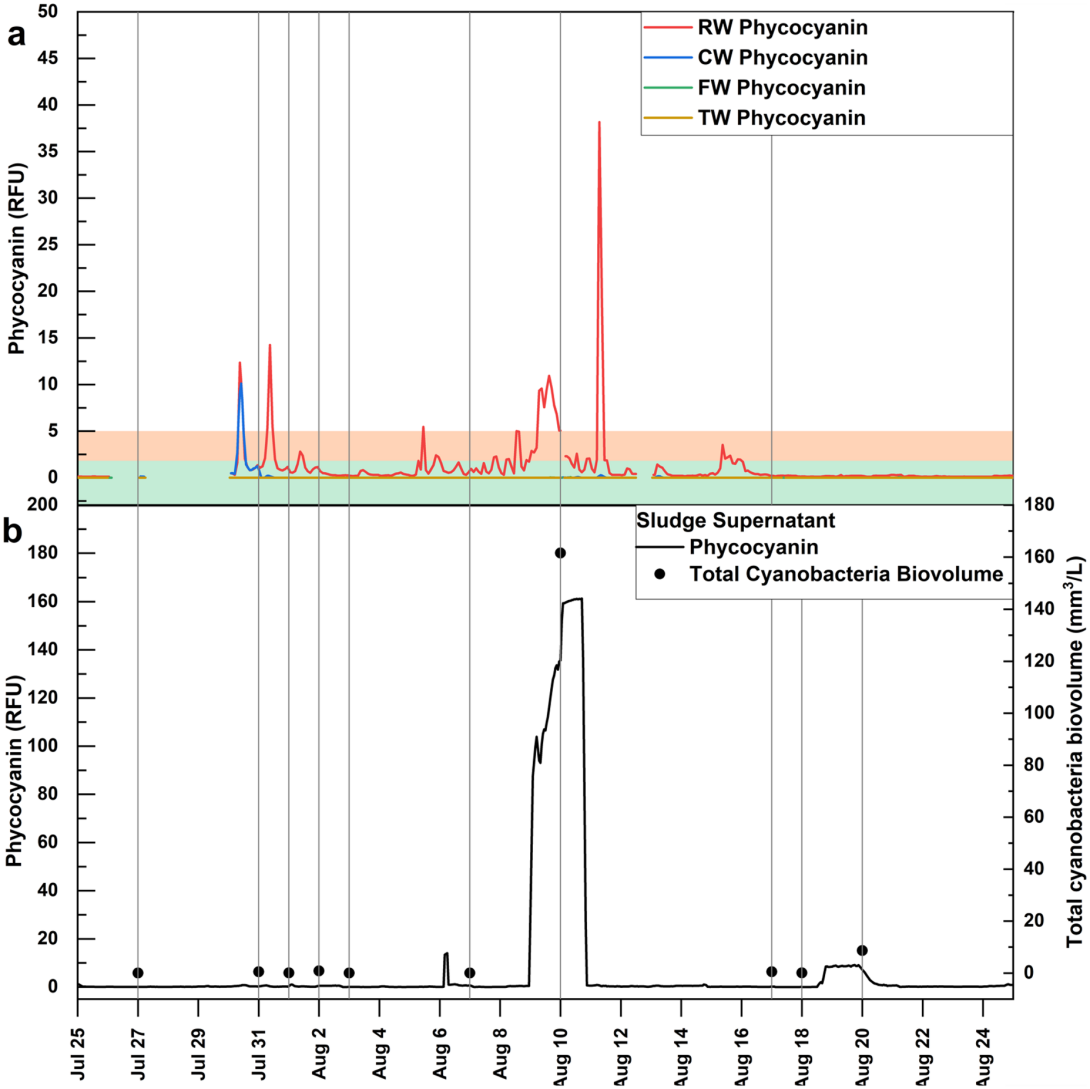
Temporal monitoring of 2-h moving average in situ phycocyanin fluorescence (RFU) of the **a** raw water (RW), clarified water (CW), filtered water (FW), and treated water (TW), **b** supernatant of sludge storage tank (Sludge Supernatant) and its total cyanobacterial biovolumes (mm^3^/L) inside the DWTP A during the bloom season of 2018. Gray vertical lines indicate when grab samplings were collected. Background colors indicate the equivalent phycocyanin RFU alert levels (1.8 and 5.0 RFU) to WHO 2021 alert levels

The 2-h moving average of phycocyanin in the RW, CW, FW, and TW at DWTP A during July and August 2018 is shown in Fig. 4a. The plant experienced high incoming cyanobacterial densities during late July and early August. On July 30 and 31 and August 5, 8, 9, 10, and 11, the probe observed remarkable increases in phycocyanin in the RW, and their phycocyanin values surpassed the alert level 2 of 5.0 RFU. Moreover, a distinct increase in phycocyanin up to 10.1 RFU on July 30 was recorded in CW (Fig. 4a).

**Fig. 5:**
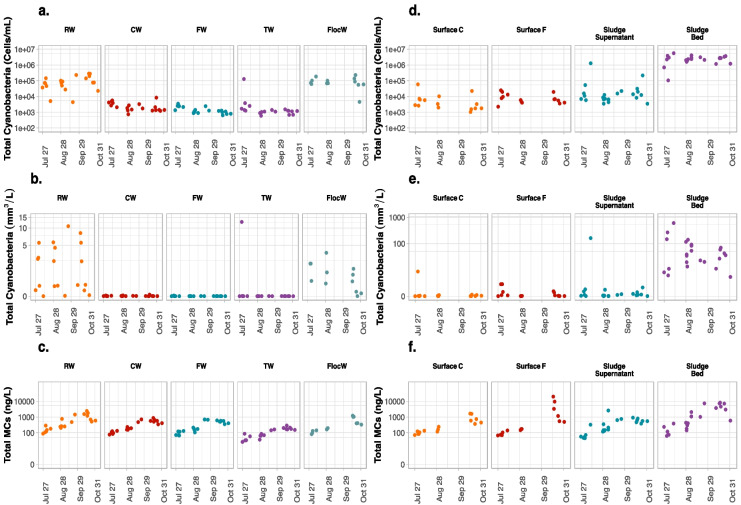
Total cyanobacterial cell counts, cell biovolumes and total MCs in water raw water (RW), clarified water (CW), filtered water (FW), treated water (TW), flocculated water (FlocW), apparent accumulation at the surface of clarified water (Surface C), apparent accumulation at the surface of filtered water (Surface F), supernatant of sludge storage tank (Sludge Supernatant), and sludge bed of sludge holding tank (Sludge Bed) of the DWTP A

While probe readings were proceeding online, grab samples were taken for taxonomic cell counts and toxin analysis (Fig. 5). The total cyanobacterial biovolume in RW on July 31 was 2.7 mm^3^/L, August 1 was 2.9 mm^3^/L, August 2 was 5.6 mm^3^/L, and August 7 was 4.3 mm^3^/L (Fig. 5b), close to or surpassed WHO alert level 2 for drinking water sources of 4.0 mm^3^/L (Chorus & Welker, 2021). In addition, on 5 days during September and October, total cyanobacterial biovolumes in RW were higher than alert level 2. However, valid probe data was lacking in parallel with the grab sampling results during September and October.

Cell counts of flocculated water taken from the flash mix were generally lower than those measured in the corresponding RW, possibly because of the impact of aggregation on the ability to enumerate or because of the impact of the coagulants used as shown recently by Le et al. (2021). During the monitoring period, the probe readings for FW and TW were lower than 0.1 RFU (Fig. 4a). In accordance, very low cyanobacterial biovolumes (lower than 0.01 mm^3^/L) were detected in FW and TW (Fig. 5b).

With regards to the total MCs in the water samples, between July 25 and August 25, total MCs in the RW remained modest, ranging from 88.4 to 286.4 ng/L. During September and October, total MCs in the RW were higher and reached up to 2456.1 ng/L on October 16. Total MCs in CW and FW were all below 900 ng/L, and in TW were below 300 ng/L during the whole sampling period (Fig. 5c).

#### Cyanobacterial cells and cyanotoxin in the surface of separation processes and in the sludge in a high-risk DWTP

The surface of separation processes and sludge in the holding tank were also sampled. Following the high incoming density of cyanobacteria arriving in the DWTP A on July 30 and the subsequent rise of cyanobacteria in CW, accumulation at the surface of these processes was noted with high cell biovolume with 7.7 mm^3^/L in Surface C and 1.9 mm^3^/L in Surface F on July 31 (Fig. 5e). During the other sampling dates, total cyanobacteria were all lower than 0.1 and 0.5 mm^3^/L in Surface C and Surface F, respectively.

An additional probe was used to monitor the cyanobacterial concentration in Sludge Supernatant inside the high-risk DWTP A. Phycocyanin probe readings started to increase at the Sludge Supernatant on August 9 and reached the highest level on August 11 at 2 h with 161.3 RFU, then decreased to 0.7 RFU on August 11 at 6 h (Fig. 4b). Like the data for the other sites, all available data regarding grab sampling in the Sludge Supernatant and Sludge Bed were compiled. Trends of cyanobacterial biovolumes result in the Sludge Supernatant conformed well with that of probe readings (Fig. 4b). When the phycocyanin level in the Sludge Supernatant reached high values on August 10, the total cyanobacterial biovolume was also found to be at the highest value with 161.5 mm^3^/L on August 10. Furthermore, the highest number of cyanobacterial biovolume was observed in the Sludge Bed with 608.7 mm^3^/L on August 10. Notably, they were 62,304 and 234,811 times greater than the RW. During the other days, total cyanobacterial biovolumes in the Sludge Supernatant were generally lower than in RW, whereas Sludge Bed samples always had higher values (1.8–1609 times) than that in RW.

The significant increase in phycocyanin readings in the Sludge Supernatant on August 10 also corresponds to the increase in cyanotoxin concentration. On August 10, total MCs in Sludge Supernatant and in the Sludge Bed reached 329.2 ng/L and 387.1 ng/L, a two-fold increase than measured in RW (183.1 ng/L). For other dates between July and August, total MCs in Sludge Supernatant and Sludge Bed were all lower than that in RW, with below 72 ng/L in Sludge Supernatant and below 120 ng/L in Sludge Bed (Fig. 5f). During September and October, on the 5 days that cell biovolumes in RW were higher than 4 mm^3^/L, the MCs in the Sludge Bed were also higher than that in RW, with up to 8288.2 ng/L in the Sludge Bed.

### Applying the probe readings to estimate the risk of cyanobacteria within two low-risk DWTPs

#### Cyanobacterial cells and cyanotoxins in the RW, CW, FW, and TW in low-risk DWTPs

**Fig. 6:**
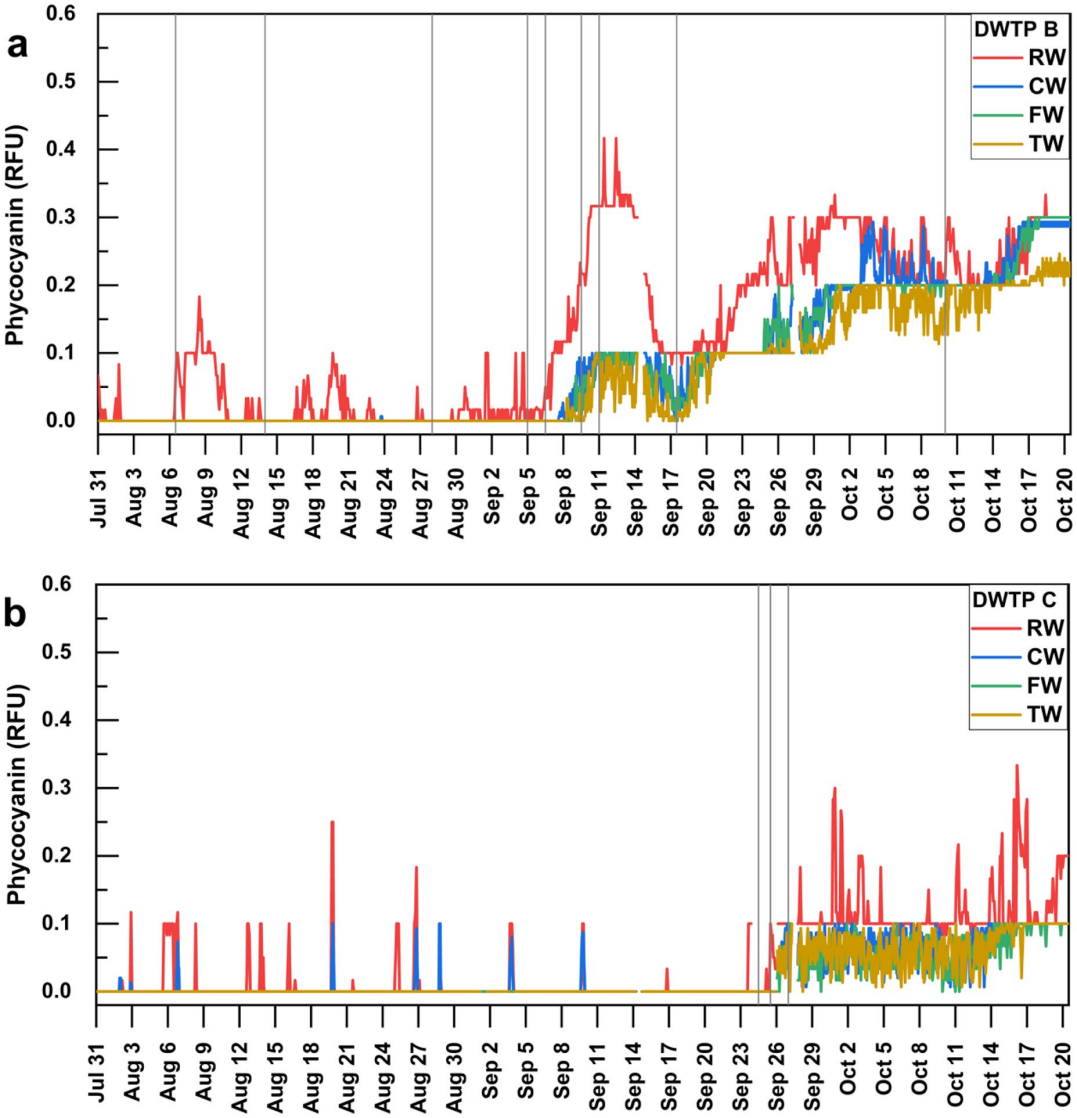
Temporal monitoring of 2-h moving average in situ phycocyanin fluorescence (RFU) of the raw water (RW), clarified water (CW), filtered water (FW), and treated water (TW) inside the **a** DWTP B and **b** DWTP C during the bloom season of 2018. Gray vertical lines indicate when grab samplings were collected

**Fig. 7:**
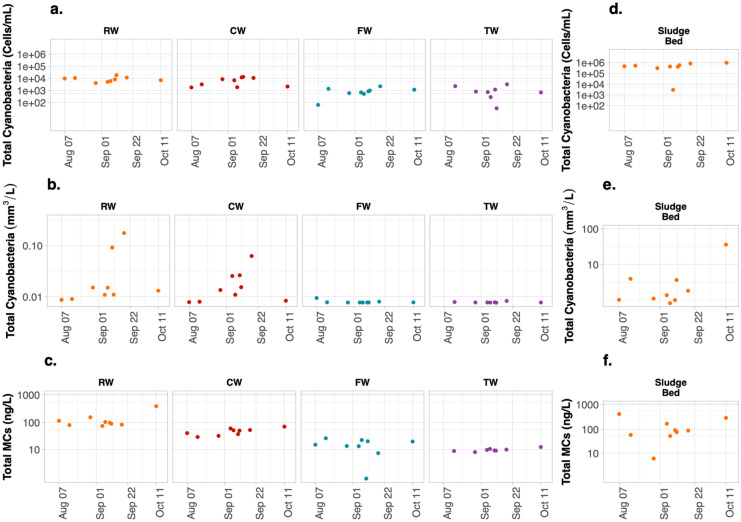
Total cyanobacterial cell counts, cell biovolumes, and total MCs at raw water (RW), clarified water (CW), filtered water (FW) treated water (TW), and sludge bed of sludge holding tank (Sludge Bed) of the DWTP B

**Fig. 8:**
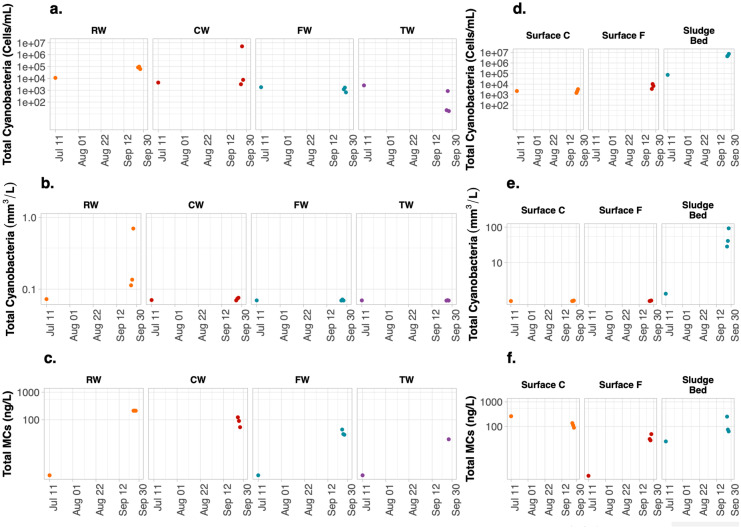
Total cyanobacterial cell counts, cell biovolumes and total MCs at raw water (RW), clarified water (CW), filtered water (FW), treated water (TW), surface of clarified water (Surface C), surface of filtered water (Surface F), and sludge bed of sludge holding tank (Sludge Bed) of the DWTP C

In situ phycocyanin probes were also used to monitor the presence of cyanobacteria at DWTPs B and C. The phycocyanin values for either of the plants were lower than 0.4 RFU during the entire monitoring period (Fig. 6). Indeed, the NOAA real-time satellite imagery did not detect any bloom at the Canadian site of Western Lake Erie (intake water for DWTP C) (NOAA, 2018). Likewise, grab sampling total cyanobacterial biovolumes with lower than 0.1 mm^3^/L for RW, CW, FW, and TW of the DWTP B and C confirmed the low phycocyanin readings (Figs. 7b and 8b). Such low values precluded any meaningful correlation relation between phycocyanin and biovolumes. DWTPs B and C provided the opportunity to monitor the low incoming level of cyanobacterial cells to the DWTPs.

#### Cyanobacterial cells and cyanotoxin at the surface of the separation processes and in the sludge in low-risk DWTPs

With regards to Surface C and Surface F, we only have results for low-risk DWTP C, the cyanobacterial biovolumes for Surface C and Surface F were all lower than that in RW, and their values were lower than 0.1 mm^3^/L (Fig. 8b, e).

Sludge in the holding tanks is the most problematic site for cell accumulation for these two low-risk plants. At DWTPs B and C, all the Sludge Bed contained total cyanobacterial biovolumes greater than that of the RW, while the other measured locations had lower values than that of RW (Figs. 7 and 8). Given the low cell flux into these two DWTPs, the observations of cyanobacteria in the Sludge Bed were surprising. The average total cell biovolume of sludge samples was 5.1 mm^3^/L at DWTP B (Fig. 7e). Even more significant accumulations were found in the Sludge Bed of DWTP C, with an average of 41.1 mm^3^/L (Fig. 8e). The largest discrepancy happened at DWTP C on September 27 when the probe showed 0.1 RFU and the total biovolume was 0.8 mm^3^/L in RW, whereas an elevated 92.8 mm^3^/L total biovolume was measured in the Sludge Bed. Higher total cyanobacterial biovolumes and counts were observed in the Sludge Bed samples from these two low-risk plants. The concentration factors reached 1828 times for DWTP B and 219 times for plant C.

Although cell biovolumes significantly increased in the Sludge Bed of the two low-risk DWTPs, total MCs were not high. On most of the days, the total MCs in the Sludge Bed were lower than that in RW (Figs. 7c and f and 8c and f). Our study did not find exceedance of drinking water standards of 1 μg/L MCs corresponding to alert level 1 (Chorus & Welker, 2021).

## Discussion

### Daily and hourly variations affect the ability to assess risk

Large daily and hourly fluctuations in phycocyanin probe readings and cell counts were observed in the RW of the high-risk DWTP. Daily fluctuation of up to 55 phycocyanin RFU was noted (Fig. 2). Higher phycocyanin readings occurred during the afternoon compared to other times of the day (Fig. 3), and statistically significant differences were found between 18 h and the other times of the day during July (Fig. S5). Zamyadi et al. (2014) found probe-based cyanobacterial cell concentrations also varied widely within a day, with the highest RFU during the afternoon. Hydrodynamic modeling of cyanobacterial blooms at Missisquoi Bay noted the cyclic nature of risk to the drinking water intake (Ndong et al., 2017). The observations are coherent with prior reports that cyanobacterial concentration can vary yearly (Bertani et al., 2017), seasonally (Gagné et al., 2018), daily (Genzoli & Kann, 2016), and hourly (Qi et al., 2018). Light availability, temperature, nutrient, wind, and shallow lake migration may explain these patterns (Aparicio Medrano et al., 2016; Ndong et al., 2014; Qi et al., 2018; Qin et al., 2018; Rousso et al., 2021). Significant efforts have been deployed to model cyanobacterial dynamics in freshwater lakes (reviewed by (Rousso et al., 2020)), and those approaches may be applied to predict future scenarios and help better the management of cyanobacterial in the DWTPs. Without the input of a dynamic measurement of incoming cyanobacteria, one event of peak cyanobacterial loading was not identified leading to the breakthrough of cyanobacterial cells and toxins as the operators failed to timely implement treatment responses.

These wide phycocyanin variations question the ability of grab samples or single probe measurements to be used against action thresholds to determine the need for corrective actions or advisories. Clearly, increases in cyanobacteria that occur in the interval between grab sampling may receive, suggesting the risk of delay or missing high cyanobacterial biomass. Routine grab sampling only provides a snapshot of the concentrations at any given time and is not indicative of cumulative or peak loadings of cyanobacteria entering the DWTP. Cumulative loadings are indicative of the amount of cyanobacteria and toxins that will be concentrated in the DWTP processes such as the clarifier and sludge, while peak loadings are indicative of periods of high risk for breakthrough. For DWTP management purpose, online monitoring or composite sampling would be better indicators of loadings.

Most guidelines advise utilities to conduct weekly sampling for cyanobacterial cell counts or biovolumes and, in some cases, toxin concentrations. For example, Australian guidance proposes a maximum sampling frequency of twice per week (Newcombe et al., 2010). For implementation reasons, utilities are likely to conduct sampling on fixed days and times, which can entirely miss short-lived and longer lasting cyanobacteria events. Sampling once or twice a week on a fixed schedule may provide misleading results. In our study, grab samplings were conducted in the morning (8 to 10 h), while cyanobacterial increased in the afternoon period at the high-risk DWTP A intake water, which indicates that peak concentrations occurring in the afternoon were unlikely to be ever measured. For example, on August 10, a maximum 2-h moving average of 10.9 RFU should warrant plant operator based on exceedance of the alert level of 5.0 RFU (Fig. 2a), while grab sampling result of 0.02 mm^3^/L was lower than the vigilance level (Fig. 5b).

Furthermore, the difference in time series trends shows the importance of the method for evaluating the risk of cyanobacteria entering the plants. The appropriate statistical treatment should be chosen in accordance with the requirement (Fig. 2). One single prominent phycocyanin peak based on raw measurements is not representative of the cyanobacteria loadings entering the plant. Moving averages for 2, 6, 12, and 18-h could detect all increases in phycocyanin for various operational purposes. In this study, 2-h moving average provides actionable information to the plant manager to be vigilant, indicating the need for immediate corrective action, such as adjusting coagulation and PAC dosages. Elevated phycocyanin values using the 6-h moving averages indicate that the bloom is persistent and may affect all separation processes over time. Daily and weekly moving averages smooth out the short-term fluctuations, which may be useful for longer term trending. In some cases, progressive increases of daily moving averages of RFUs are indicative of upcoming large peaks of phycocyanin concentration. For example, the daily moving average started to increase on July 27 and August 8 and occurred prior to the large peaks on July 30 and August 11 based on raw measurements (exact time). Similar results were found for weekly moving averages which provided increased signals prior to the raw measurements. However, the weekly moving average consistently underestimated the phycocyanin readings and cannot detect the peaks.

### Relationships between phycocyanin RFU and total cyanobacterial biovolumes

Conversion of phycocyanin fluorescence readings to total cyanobacterial cell counts and/or biovolumes is required when using phycocyanin estimations to relate readings to cyanobacterial risks in a water treatment plant. Numerous studies have provided a strong correlation between phycocyanin readings and cyanobacterial cell counts/biovolumes for field-collected samples (Cotterill et al., 2019; Kong et al., 2014; Thomson-Laing et al., 2020; Zamyadi et al., 2014). However, only a few studies have assessed the statistical treatment of phycocyanin readings to improve the interpretation of grab sample cyanobacteria biovolumes. Genzoli and Kann (2016) established different statistics to characterize phycocyanin patterns in the Klamath River in the western United States to predict the risk of toxins in source water. The authors compared taxonomic cell counts to YSI 6600 phycocyanin probe readings considering hourly means, daily means, daily 10% trim means, minimums daily, maximums daily, 25th and 75th quantiles of daily, daily medians, and moving averages (4, 8, and 14 days). Although the authors noted similar correlation coefficients (ranging from 0.69 to 0.77) between cell counts and all statistics phycocyanin readings, low grab sampling frequency (weekly or biweekly) could hardly represent the phycocyanin conditions. These findings apply to the prediction of risk at the source. In our study, only the 2-h moving average was best correlated with the total cyanobacterial biovolumes (Fig. S6). We consider the relative use of different statistics of the probe readings to predict the level of challenge to the separation processes in a DWTP. A shorter 2-h moving average was indicative of the incoming loadings. But for clarified and filtered water, a correlation cannot be established between the biovolumes and the RFU probe readings, because most readings were below the detection limit.

Although not the focus of this study, fluorometric interferences (e.g., temperature, presence of eukaryotic algae, and DOM), light exposure, and turbidity are the factors that can influence the phycocyanin readings (Choo et al., 2019; Ma et al., 2022; Rousso et al., 2021; Symes & Van Ogtrop, 2016). In our study, the extent of such interference may have influenced the results was low. With regards to DOM and temperature, as the sampling was conducted between July and October, a sudden change of DOM level was unlikely to happen, and the maximum temperature difference in the raw water of the studied DWTPs was 5 °C (data not shown). The impact of eukaryotic algae in our study is unlikely to be large, as the highest chlorophyll-a was in the raw water of the high-risk DWTP A with 4.5 RFU (data not shown), and the average chlorophyll-a at the other sites of three studied DWTPs was all approaching 0. Almost no light exposure was applied to the in-plant samples. Turbidity over 50 NTU can make probe measurement ineffective (Bowling et al., 2013); but in our study, for the raw water of DWTP A, there was only one point (5 min on August 16) with turbidity over 50 NTU. It was also reported that the nutrients could have an effect on phycocyanin production of a microalgae (Wicaksono et al., 2019). However, in our study, the nitrate and phosphate concentrations of all the samples were ranging from 0.04 to 0.36 mg/L and 0 to 0.05 mg/L, respectively (data not shown), much smaller than that of 12.5 to 100 mg/L and 0.25 to 2 mg/L in the study of Wicaksono et al. (2019). So the interference of nutrients could also be neglectable.

Besides the interference from the environment, different species present and colony formation can also contribute to the uncertainty of phycocyanin readings (Chang et al., 2012; Hodges et al., 2018; Rousso et al., 2022). Cyanobacterial density evaluated as biovolumes in the three studied DWTPs displayed considerable variability within each site over the course of sampling (data not shown). Ma et al. (2022) provided data concerning the various species present in the RW in DWTP A. Such variations of cyanobacterial biovolumes and species were also noted in other studies during DWTP monitoring campaigns (Jalili et al., 2021; Zamyadi et al., 2013a). Nevertheless, this interspecific difference in phycocyanin readings could be reduced by converting cell counts to biovolumes (Ma et al., 2022; Macário et al., 2015).

### Mapping the location of cyanobacterial cells in the high and low-risk DWTPs

In high-risk DWTP A, high numbers of total cyanobacterial biovolumes on the Surface C reached 7.7 mm^3^/L as observed on July 31 (Fig. 5e). The Surface C accumulation may lead to a risk of cyanobacterial cells passing into the CW as clarified water is collected on top of these clarifiers (Zamyadi et al., 2013b). Indeed, on that day, we observed relatively high phycocyanin levels in the CW with up to 1.2 RFU. Furthermore, this greater breakthrough resulted in higher total cyanobacterial biovolume on the Surface F with 1.8 mm^3^/L. However, probe readings and grab sampling taxonomic results show that cyanobacteria did not pass through into FW during all events monitored. The low cyanobacteria level in FW may due to adequate capture by the filters or by the dilution of the limited accumulation of CB in the volume of water over the filters (Zamyadi et al., 2013b).

Regarding the two low-risk DWTPs, cell accumulation was not noted on the surface of clarifiers (Surface C) or filters (Surface F). Almuhtaram et al. (2018) and Zamyadi et al. (2013b) also studied other low-risk DWTPs with cyanobacterial concentration in the RW lower than 1000 cells/mL. Almuhtaram et al. (2018) also reported no accumulation at the surface of clarifiers and filters in four DWTPs, while Zamyadi et al. (2013b) reported cyanobacterial scums with a concentration of up to 1.5 × 10^6^ cells/mL in one of two plants. Pre-oxidation was identified as an approach to damage to cyanobacterial cells, affecting the cells’ integrity and buoyancy, thus decrease the cyanobacterial accumulation at the surface of clarifiers and filter (Moradinejad et al., 2019). Indeed, pre-chlorination was implemented for the intake water of the DWTPs in our study and Almuhtaram et al. (2018), while the DWTP in Zamyadi et al. (2013b) was not equipped with pre-oxidation.

As expected, the concentrated particles in the sludge in the high and low-risk DWTPs led to higher cyanobacterial biovolumes in the Sludge Bed than in the RW. Despite the higher concentration of cyanobacteria in the sludge, typically contained lower levels of total MCs than in the RW, except for the worst-case scenarios for the high-risk DWTP A. These observations are in agreement with Jalili et al. (2021) who reported low MCs in Sludge Bed (below 281 ng/L) in the same DWTP A, although the Sludge Bed experienced 3–31 times higher cell numbers as compared to RW. Similar patterns of MCs were also observed in the low-risk DWTPs where MCs cell quota in the Sludge Bed of 0.05 pg/cell was around 70 times lower than in the RW of 3.3–4.2 pg/cell (Almuhtaram et al., 2018), indicative of elevated cell numbers yet low MCs in the Sludge Bed. Lower levels of MCs in the sludge can result from the degradation and absorption on PAC as shown by Jalili et al. (2021). Although the high cyanobacterial densities in the sludge did not always represent a considerable risk of toxins, several investigations have shown that cyanobacterial cells may survive and even proliferate at least 10 days in the sludge storage tank and release cyanotoxins (Jalili et al., 2022; Pestana et al., 2016). The presence of nutrients could influence the dynamics (Dreyfus et al., 2016; Pestana et al., 2016). We also observed high total cyanobacterial biovolumes and total MCs in the Sludge Supernatant when the cell concentrations in the Sludge Bed were high. Therefore, the stored sludge should be considered for cyanotoxin release source when (1) recycling the sludge supernatant to the head of the DWTP and (2) discharging the sludge bed to the wastewater treatment plant.

## Conclusion

- Phycocyanin readings in the raw water fluctuated up to 93-fold (from 0.6 to 55.8 RFU) within 24 h. The wide daily and hourly variations in phycocyanin readings show the dynamic of cyanobacterial risk during a bloom period
- Monitoring for risks at the raw water or across the plant cannot be achieved using daily grab sampling, as it could only give a snapshot of the cyanobacterial concentrations at a given time. And most importantly, grab sampling cannot provide an estimate of the dynamic flux of the cyanobacteria entering a DWTP
- A 2-h moving average of the phycocyanin readings was proposed to improve the interpretation of phycocyanin signal trends and guide treatment response, avoiding responding to peaks of very short duration
- An in situ phycocyanin probe was applied to successfully detect high cyanobacterial biovolumes entering a high-risk DWTP and the subsequent removal of cyanobacteria by clarification, filtration and chlorination
- Significant cell accumulations will occur in the sludge in both high and low-risk DWTP. Such accumulations were not always associated with elevated MCs.

### Supplementary Information

Below is the link to the electronic supplementary material.

Supplementary file1 (DOCX 4600 kb)

## Data Availability

The datasets generated during and/or analyzed during the current study are available from the corresponding author on reasonable request.
