# Supplementary material for: Monitoring of cyanobacterial breakthrough and accumulation by in situ phycocyanin probe system within full-scale treatment plants
Source: Environ Monit Assess. 2023 Aug 17;195(9):1042. doi: 10.1007/s10661-023-11657-0 (PMC10435606; doi:10.1007/s10661-023-11657-0)
Supplement: Supplementary file 1 — Supplementary file1 (DOCX 4600 kb) [file 10661_2023_11657_MOESM1_ESM.docx]

Article

**Monitoring of cyanobacterial breakthrough and accumulation by *in situ* phycocyanin probe system within full-scale treatment plants**

Liya Ma ^1,*^, Juan Francisco Guerra Maldonado ^1^, Arash Zamyadi ^2^, Sarah Dorner ^1^, and Michèle Prévost ^1^

^1^ Department of Civil, Geological, and Mining Engineering, Polytechnique Montreal, Montreal, Quebec, H3C 3A7, Canada

^2^ Department of Civil Engineering, Monash University, Clayton Campus, Melbourne, Australia

***** Correspondence: [liya.ma@polymtl.ca](mailto:liya.ma@polymtl.ca)

# **Supplementary Materials**


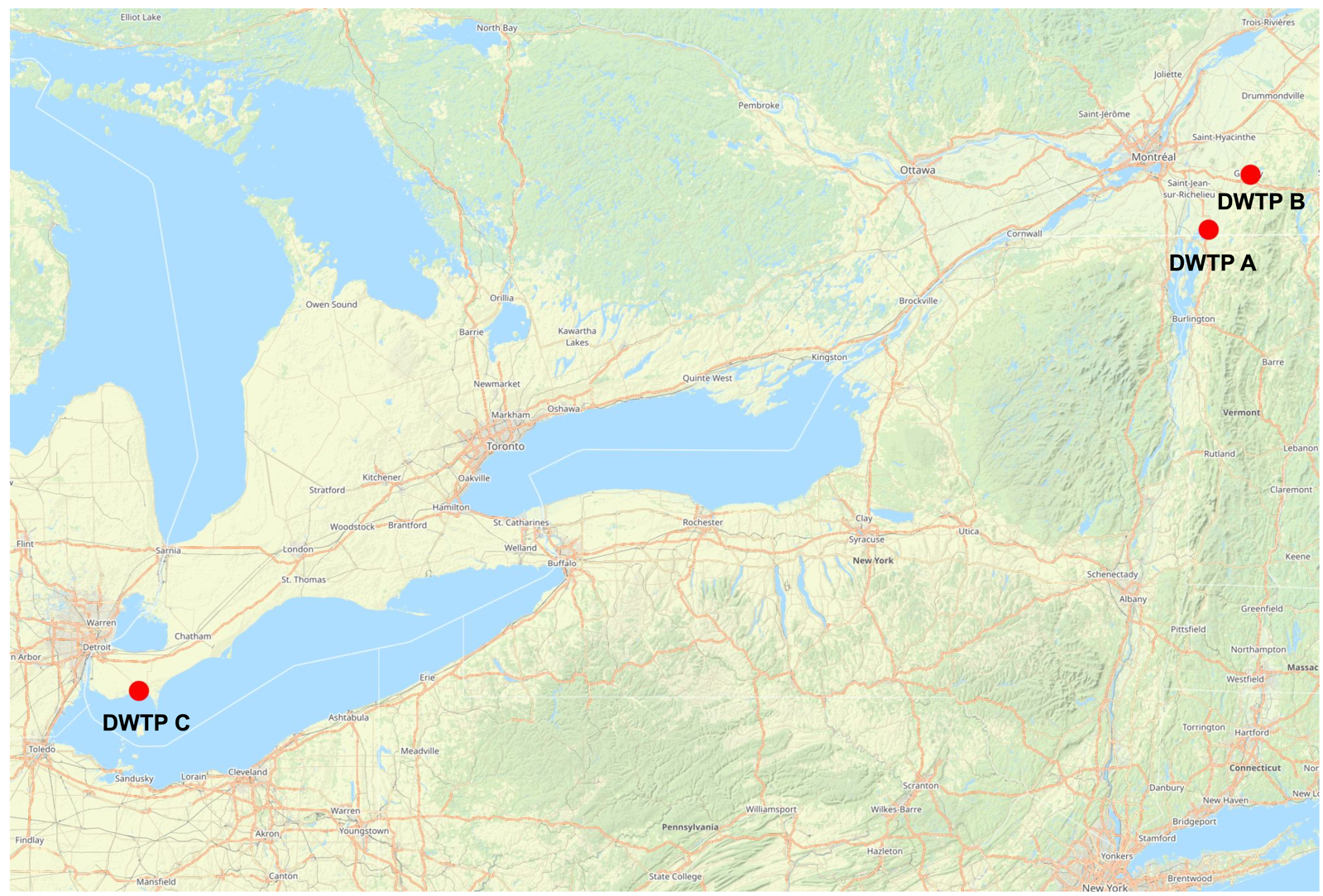


**Fig. S1** The location of the studied three DWTPs: A and B are located in southern Quebec, and C is located in southwest Ontario


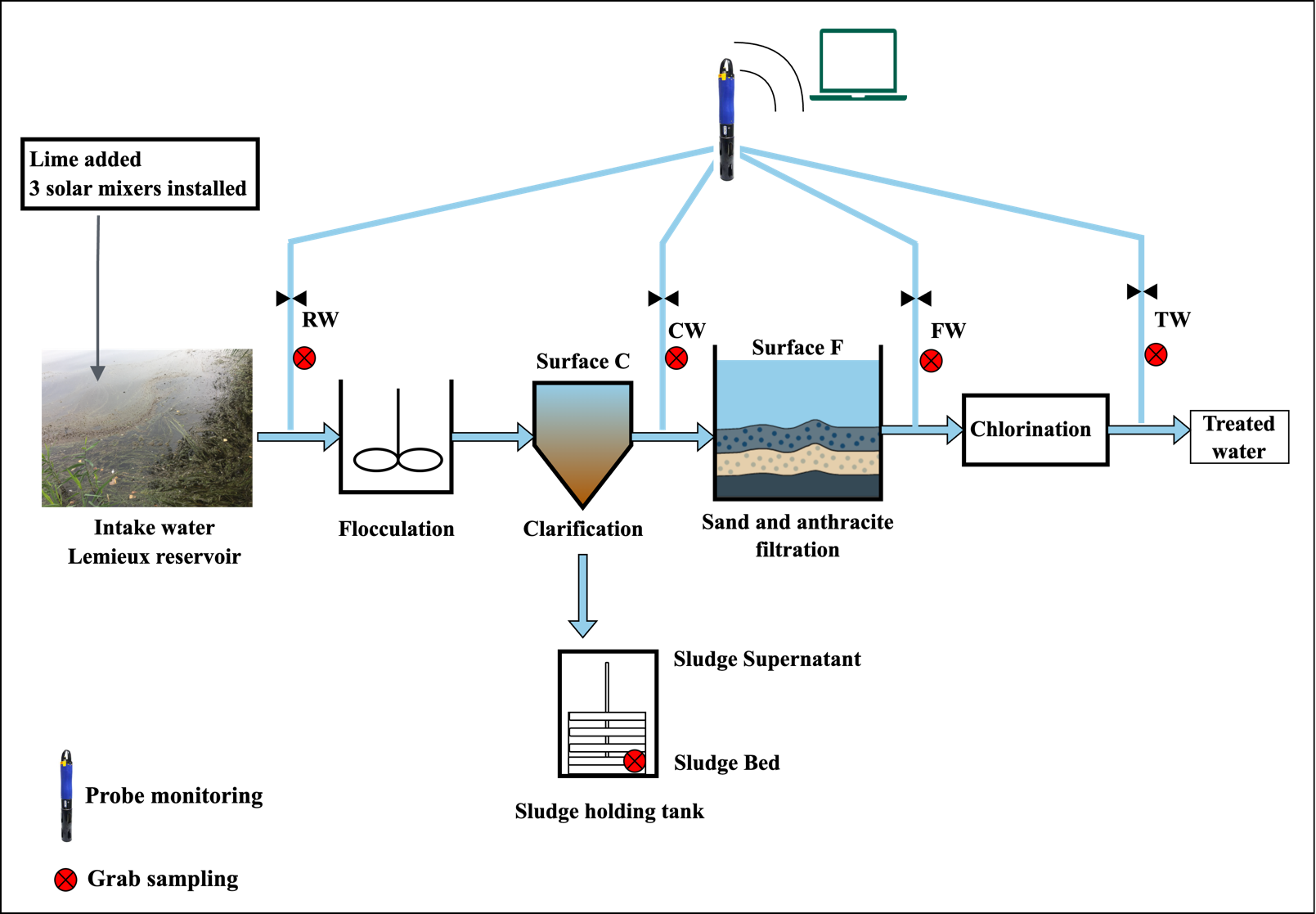


**Fig. S2** Schematic of the treatment processes of DWTP B, grab sampling and probe monitoring locations. The water intake is from Lemieux reservoir. Sampling points are indicated by: raw water (RW), clarified water (CW), filtered water (FW), treated water (TW), and sludge bed of sludge holding tank (Sludge Bed)


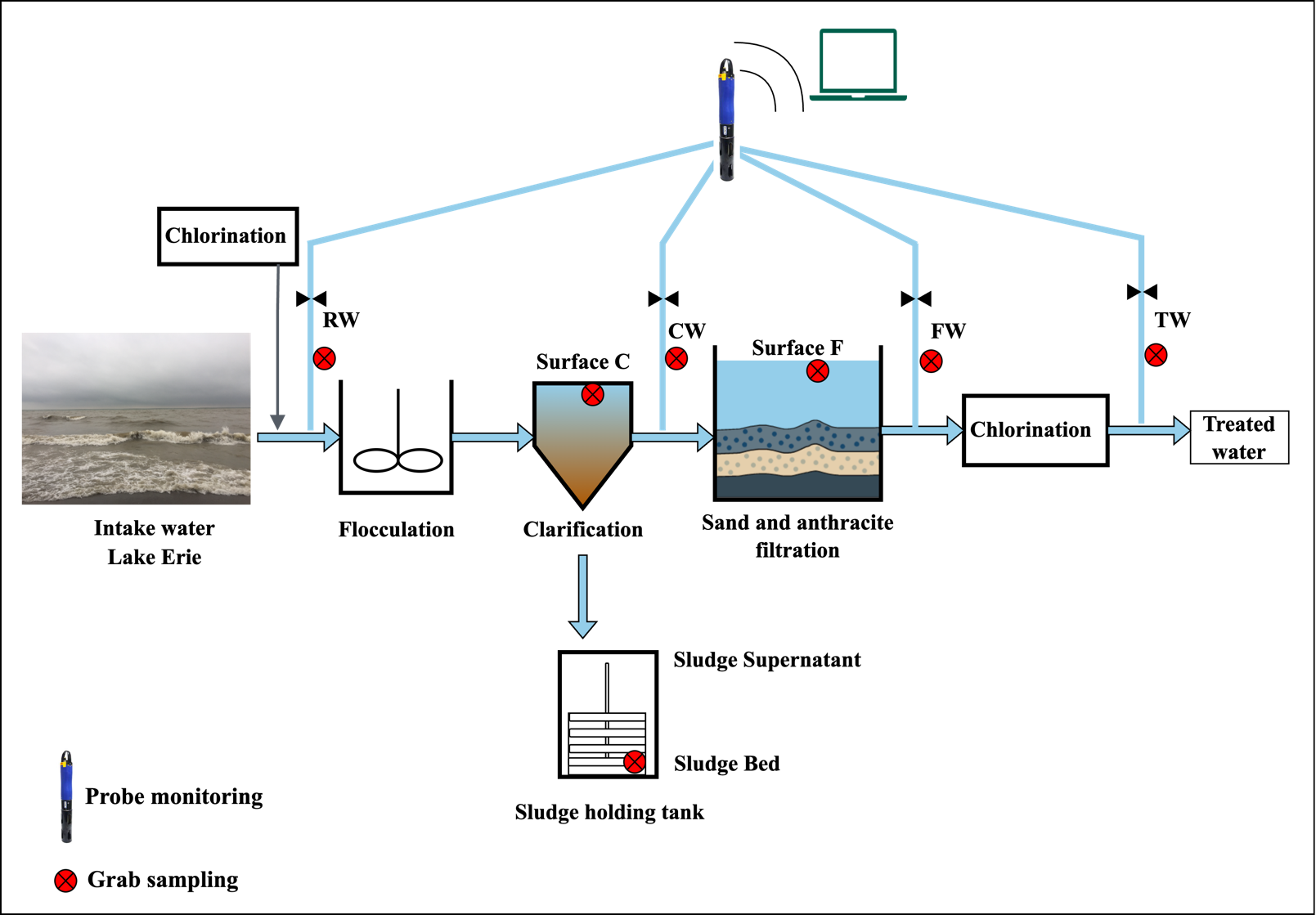


**Fig. S3** Schematic of the treatment processes of DWTP C, grab sampling and probe monitoring locations. The water intake is from Lake Erie. Sampling points are indicated by: raw water (RW), clarified water (CW), filtered water (FW), treated water (TW), surface of clarifier (Surface C), surface of filter (Surface F), and sludge bed of sludge holding tank (Sludge Bed)


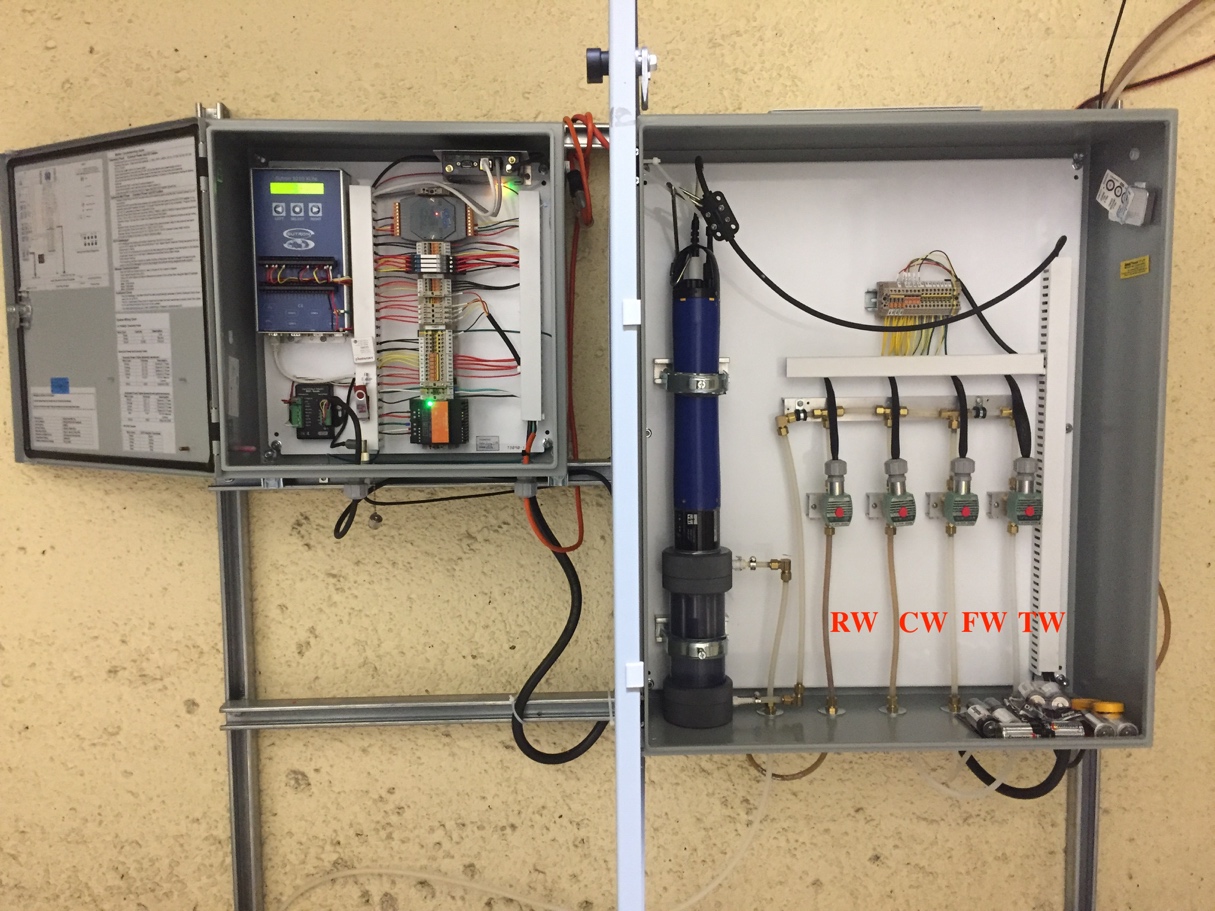


**Fig. S4** Example of YSI EXO2 probe data collection system at the studied three DWTPs. Sampling points are indicated by: raw water (RW), clarified water (CW), filtered water (FW) and treated water (TW)


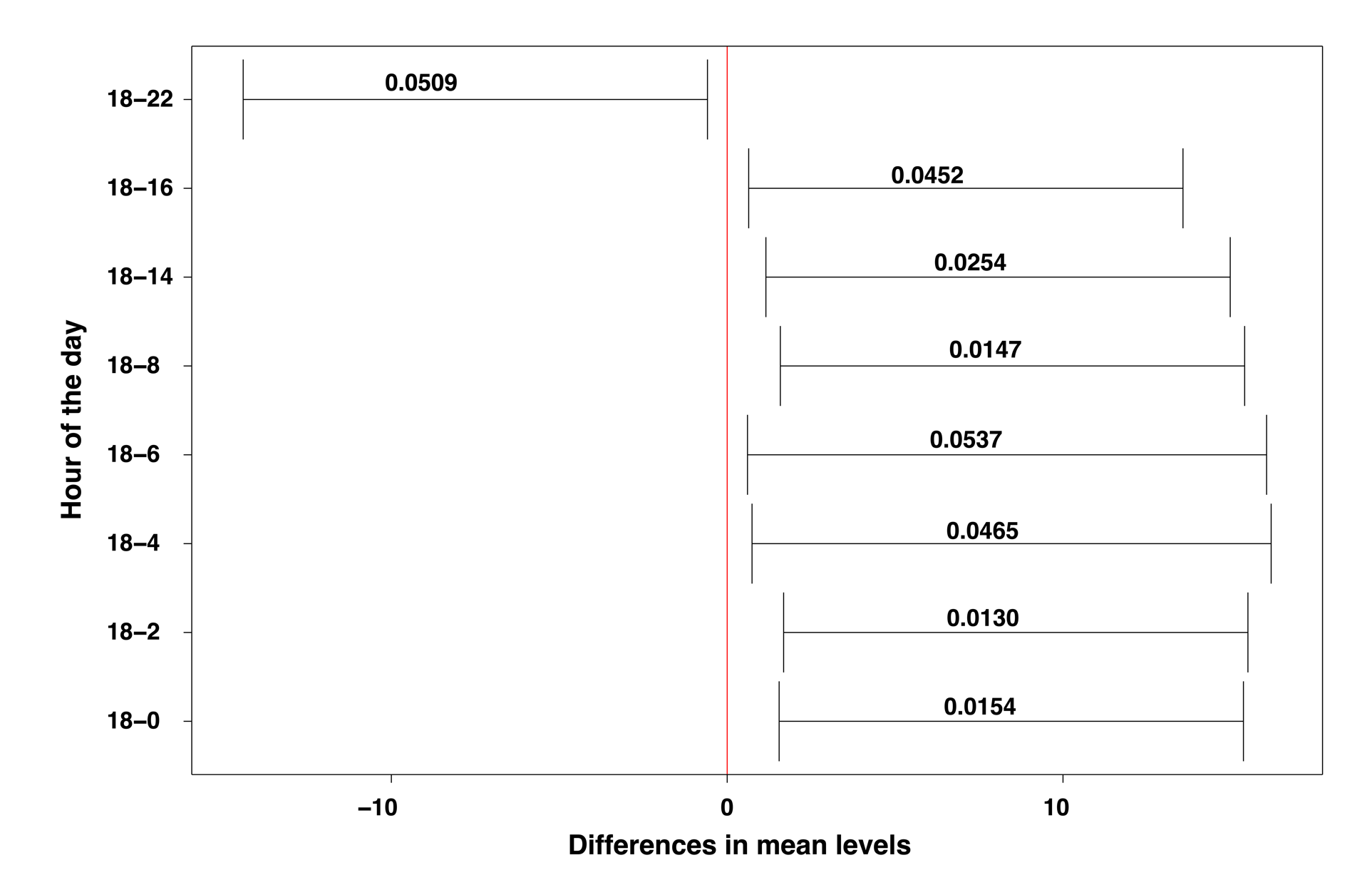


**Fig. S5** Post-hoc analysis (Tukey’s HSD) of the phycocyanin RFU readings during July following ANOVA with group variables of 18 h and the other measured times (significance level α = 0.1). Numbers in the graph indicate the adjusted *p* value


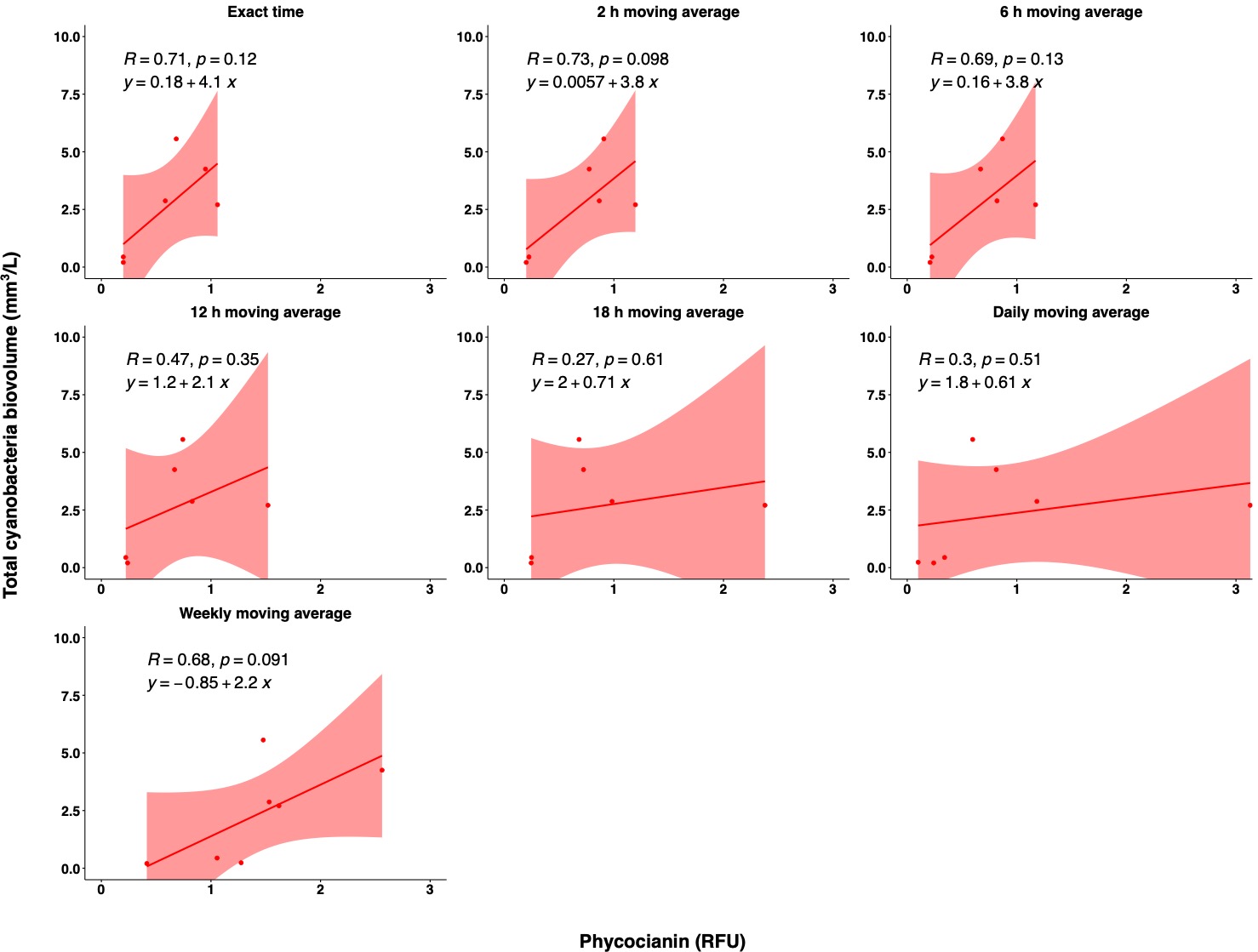


**Fig. S6** Correlations between phycocyanin (RFU) YSI EXO2 probe readings computed by different time series analysis (exact time, 2, 6, 12 and 18-hour moving averages, daily moving average and weekly moving average) and total cyanobacterial biovolume (mm^3^/L) from grab samples of the raw water of DWTP A

**Table S1** Details of treatment process characteristics of the studied three DWTPs

| **Parameters** | **A** | **B** | **C** |
| --- | --- | --- | --- |
| **Source water entering the plant intake** | Missisquoi Bay | Yamaska Reservoir | Lake Erie |
| **Hydraulic retention time** | Sludge blanket clarifier: 1.2–2.9 h  Filter: 0.7–1.6 h | Sludge blanket clarifier: 1.43–2.54 h  Filter: 0.28–0.56 h | Clarifier: 1.7 h (average)  Filter: 0.1 h (average) |
| **Pre-treatment prior to coagulation** | N/A | Lime: 5.7–13.08 mg/L | 0.3–0.5 mg/L total chlorine as residuals |
| **Coagulant** | 0.08 L PAX/L (average) | ALS-2260 (Alufer) and PIX-312 (Ferric Sulfate): 42.64–83.65 µg/L | Hyperion 1090: 15–20 mg/L |
| **Polymer** | N/A | Superfloc C-492PWG: 0.12 mg/L | Magnafloc LT 22: 0.1–0.15 mg/L |
| **PAC**  **(powdered activated carbon)** | 3.7 mg/L (average) | 9.95–14.94 mg/L | 3–4 mg/L |
| **Post chlorination** | 0.04–3.08 mg/L total chlorine as residuals | NaOCl:  0.99–1.06 mg/L | 1.7 mg/L total chlorine as residuals |
